# Staged management of a giant cardiac hydatid cyst: a case report

**DOI:** 10.1186/s12879-018-3599-2

**Published:** 2018-12-27

**Authors:** Jadranka Separovic Hanzevacki, Hrvoje Gasparovic, Vlatka Reskovic Luksic, Zvonimir Ostojic, Bojan Biocina

**Affiliations:** 1 Department of Cardiovascular Diseases, School of Medicine, University Hospital Centre Zagreb, Kispaticeva 12, 10 000 Zagreb, Croatia; 2 Department of Cardiac Surgery, School of Medicine, University Hospital Centre Zagreb, Zagreb, Croatia

**Keywords:** Cardiac, Hydatid cyst, Echinococcosis, Surgery

## Abstract

**Background:**

We report on a 21-year-old patient with a giant symptomatic hydatid cyst of the interventricular septum, to whom a staged management approach was employed. Induction medical therapy led to a reduction in the size of the cyst, which was then completely removed via surgical excision.

**Case presentation:**

A 21-year-old male Caucasian, with main complaints of fatigue and palpitations, was referred to our Centre due to a cystic formation in his left ventricle. The workup consisted of transthoracic echocardiography and cardiac magnetic resonance, which revealed a huge hydatid cyst in an active stage of disease, occupying the basal and mid part of the interventricular septum. Due to the size of the lesion and lack of viable myocardium in the affected area, the patient was declared inoperable and medical therapy was initiated. Serial echocardiography revealed a significant reduction in the size of the lesion and degradation to transitional and inactive stage, after which successful surgical excision of the cyst was performed. In the course of the medical treatment, the patient experienced sustained ventricular tachycardia causing loss of consciousness, which did not reoccur after surgical excision.

**Conclusion:**

Medical therapy can result in the degradation of a giant heart hydatid cyst, enabling surgical excision. Heart hydatid cyst can lead to potentially lethal arrhythmia irrespective of its size and stage, which does not reoccur after successful surgical excision.

**Electronic supplementary material:**

The online version of this article (10.1186/s12879-018-3599-2) contains supplementary material, which is available to authorized users.

## Background

Echinococcosis is a protozoal infection usually affecting people living in rural, sheep-raising areas. Although it is currently rare in developed Western countries, globalization, increasing travel opportunities and migrations harbor the potential to raise its prevalence. Infection most commonly affects the liver and lungs (70 and 20% of cases, respectively). Cardiac involvement is observed in less than 2% of cases, with the primary infection of the heart being extremely rare (< 0.2%) [1]. It can be found in any part of the myocardium, but it is most commonly located in the free wall of the left ventricle (LV). Interventricular septal (IVS) involvement is observed in 4% of cases [2]. Most patients are asymptomatic. Dyspnea, palpitations and chest pain [3] occur in a minority of patients. The preferred management option remains complete surgical excision, due to the risk of cyst rupture. The latter is associated with a high mortality risk, secondary to acute pulmonary hypertension, peripheral embolization and anaphylactic reactions [4]. The cornerstones of medical therapy are albendazole and praziquantel. A typical diagnostic algorithm includes echocardiography and computed tomography or cardiac magnetic resonance (CMR) [5]. Although ultrasound is a cornerstone of diagnosis, magnetic resonance with heavily T2-weihgted series is preferable to computed tomography for extrahepatic echinococcosis [6]. Either way, imaging is used to classify lesions into six types, which define three stages of the disease; active, transitional and inactive [7]. Management options are affected by disease staging, as inactive cysts have the lowest chance of rupture [8]. Most often, after imaging, serology testing (Western blot and enzyme-linked immunosorbent assay) is performed to confirm the diagnosis. Definitive confirmation is based on pathohistological examination of intraoperative specimens.

The aim of this case report is to present successful staged management approach in the treatment of a giant hydatid cyst, in which medical therapy led to cyst degradation, enabling surgical excision.

## Case presentation

A 21-year-old male Caucasian was referred to our Center due to a cystic formation in his LV. He is a student and lives in a house with his parents, in a rural area of the country, but they do not work in agriculture. He complained about fatigue and weakness with exertion, as well as palpitations and blurred vision. He was completely asymptomatic up until 6 months prior to his current presentation. His physical examination was unremarkable. His high sensitive troponin T and N-terminal -pro-brain natriuretic peptide were normal. His 24-h electrocardiogram was notable for non-sustained ventricular tachycardia and biphasic T waves.

**Fig. 1:**
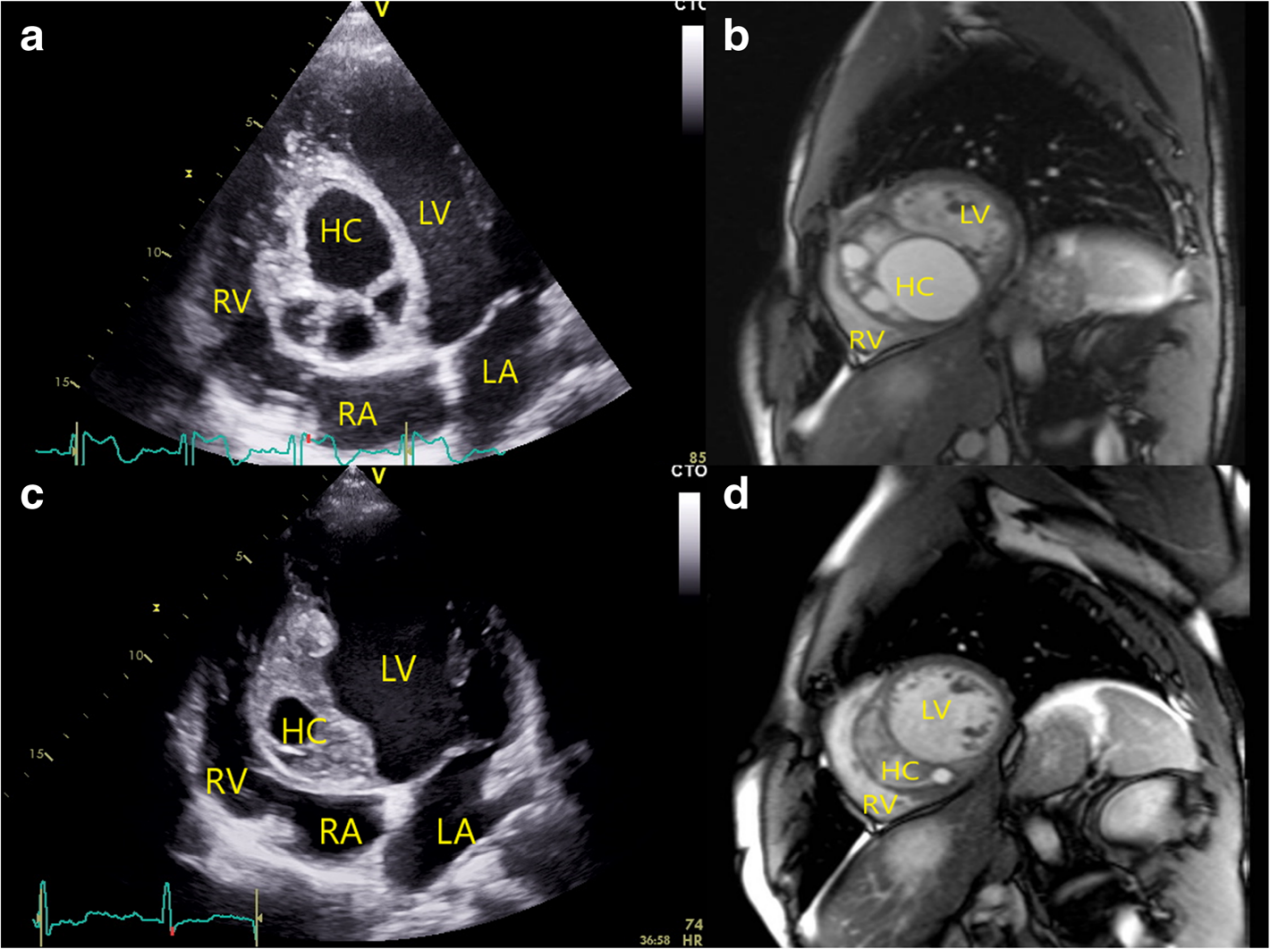
Giant hydatid cyst in the interventricular septum; (**a**) 2D transthoracic echocardiography, apical 4-chamber view and (**b**) cardiac magnetic resonance, short axis view - showing largest daughter cyst protruding in the left ventricle cavity while smaller daughter cysts protrudes in the right ventricle. **c** 2D transthoracic echocardiography, apical 4-chamber view and (**d**) cardiac magnetic resonance, short axis view – after 8 weeks of albendazole therapy, showing degradation of daughter cysts with the consequent reduction of the total hydatid cyst size. (HC = hydatid cyst, LA = left atrium, LV = left ventricle, RA = right atrium, RV = right ventricle)

Transthoracic echocardiography (TTE) revealed a multivesicular, septated cystic formation with a thin outer wall, within the basal and mid part of the IVS. The largest daughter cyst measured 6.5 × 6 cm and protruded into LV cavity (Fig. 1a). All the other daughter cysts protruded into the right ventricle (RV) (Fig. 1a). Mild inflow obstruction of the RV was present. No connections between the cysts and blood flow were observed on continuous-wave Doppler and color-flow mapping. Further workup consisted of CMR, which corroborated the TTE findings. Total lesion size was 8.2 × 7.6 × 6 cm. Residual myocardium was present only in the apical segments of the IVS, while none was observed in the affected area (Fig. 1b). Thoracic and abdominal imaging revealed multiple hepatic cysts. Serologic confirmation of echinoccocal infection was performed with Western blot and enzyme-linked immunosorbent assay. Based on its imaging characteristics, the cystic pathology was described as a type 2 active lesion.

Technical challenges from a surgical perspective included total removal of the cyst without disseminating its contents, as well as a potentially complex reconstruction of the IVS. Consequently, medical therapy with albendazole (dose: 15 mg/kg) was initiated. Bisoprolol and amiodarone were administered due to ventricular arrhythmia. Over the next 8 weeks, the patient was monitored closely and serial TTE examinations revealed a great reduction in total cyst size (Fig. 1c). Downsizing was observed in all daughter cysts, with practically complete degeneration of the largest daughter cyst. Furthermore, its content was denser and more compact, just as its outer wall was thicker. These findings were confirmed on the CMR – the size of the hydatid cyst was now 6.8 × 2.8 × 5.1 cm and the outer layer was fibrotic (Fig. 1d). Based on these findings the cyst was then classified as transitional, borderline inactive. We found neither imaging nor clinical evidence of cyst rupture. Nevertheless, full body checkup was repeated and no signs of spreading infection or embolism were observed. Praziquantel, in the total dose of 50 mg/kg, divided into 3 doses per day, during 14 days, was added to the therapy, after which surgery was scheduled. During this period the patient experienced an episode of sustained ventricular tachycardia accompanied by loss of consciousness.

**Fig. 2:**
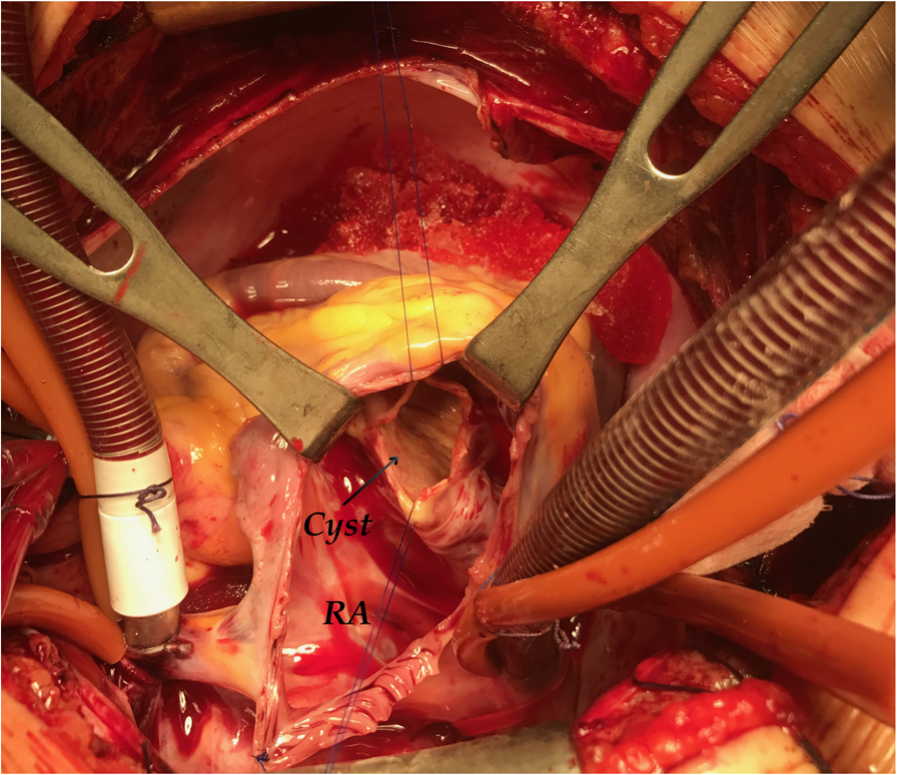
Intraoperative view of the hydatid cyst through the tricuspid valve. The arrow points to the surgically created orifice of the cyst. (RA = right atrium)

**Fig. 3:**
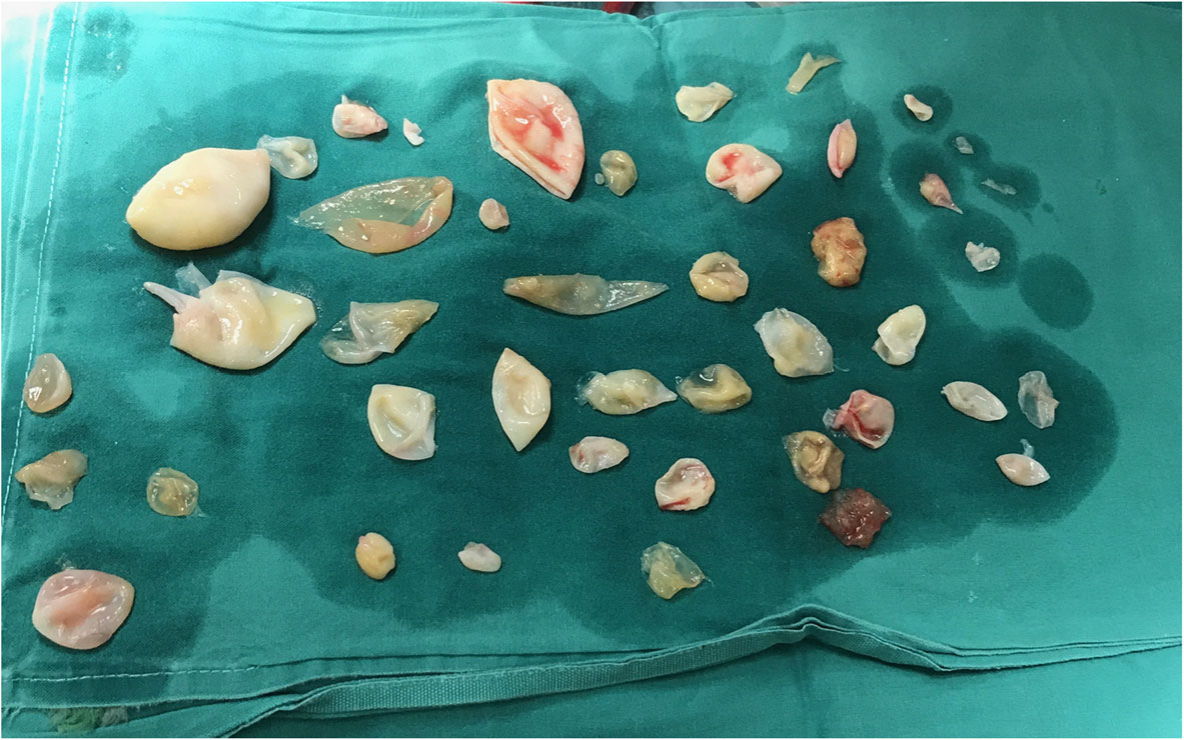
Multiple daughter cysts removed from the primary hydatid cyst

The surgical procedure consisted of several steps. After the initiation of cardiopulmonary bypass and cardioplegic arrest, a right atriotomy was performed and the pathology could be seen through the tricuspid valve. A protrusion within the IVS was clearly visible, and corresponded to the imaging data. Initially, a purse string was placed in the fibrous wall of the cyst. Then, controlled aspiration of its fluid content was performed, which effectively reduced the size of the cyst, as well as the tension within it. The opening in the cyst was then enlarged in order to gain access to the remainder of its contents (Fig. 2). The cyst was filled with multiple smaller cysts suspended in a more liquid substrate. All of these were removed, taking care not to disseminate their contents (Fig. 3). Upon the removal of the substrate, pericyst was packed with 10% sodium chloride. Sponges impregnated with the solution were left in place for 15 min. The inner layer of the pericyst was destroyed with a combination of mechanical abrasion and chemical agents. The free edges of the pericyst were then resected, and the integrity of the IVS checked. The operation was completed in the standard fashion. Intraoperative transesophageal echocardiography was used to confirm IVS integrity and tricuspid valve competence. Pathological and microbiological analysis of the intraoperatively collected material confirmed the initial diagnosis.

**Fig. 4:**
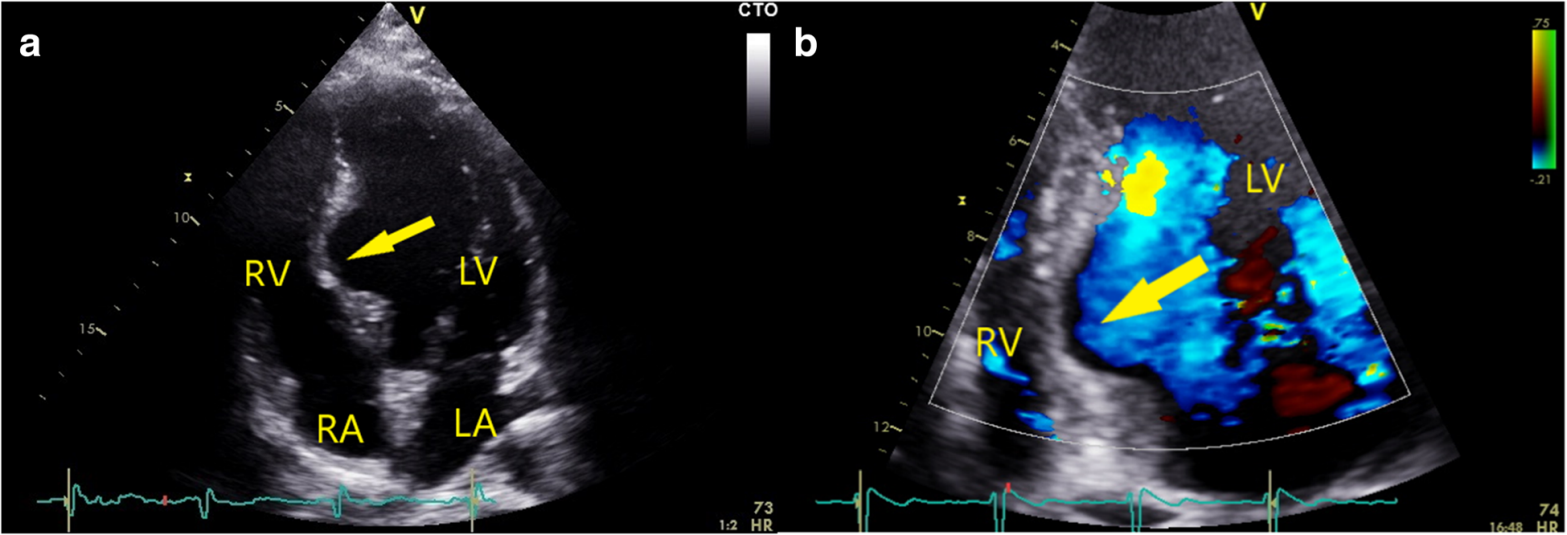
2D transthoracic echocardiography, apical 4-chamber view of the interventricular septum 6 months after the surgical procedure; (**a**) the arrow points to the residual aneurysm of the basal part of the intraventricular septum. **b** zoomed-in view of the interventricular septum with color flow Doppler showing no signs of interventricular shunt. (LA = left atrium, LV = left ventricle, RA = right atrium, RV = right ventricle)

The patient had an unremarkable postoperative course. Postoperative TTE demonstrated normal size and function of both ventricles with an aneurysm of the basal IVS but with no signs of an interventricular shunt (Fig. 4). Medical therapy with albendazole was continued for three additional cycles of 28 days each, with 14-day intervals between 28-day cycles. Six weeks later, clinical and TTE examination results were unchanged. The hepatic lesions were addressed after the cardiac surgical procedure. At 6-month follow-up the patient remains well and free of disease recurrence or ventricular arrhythmia (Additional file 1).

## Discussion and conclusion

The case presented here stands out for several reasons. The size and location of the cyst within the interventicular septum provided a distinctive management challenge. Our patient also had RV inflow obstruction compounded by ventricular tachycardia. Hydatid cysts within the heart have only rarely been found to exceed 5 cm in size. Only one surgically treated giant IVS hydatid cyst has been described so far, which resulted in death due to RV failure [9].

We adopted a staged approach in the management of this lesion. Presurgical medical management was also employed in order to reduce the activity of the target pathology and to reduce the likelihood of echinoccocus dissemination. During the course of antimicrobial treatment, we saw a progressive diminution in the size of the cyst. A reduction in the size of a hydatid cyst is an exceptionally rare event, and one which, to the best of our knowledge, has only been described once before [10]. We opted to exploit this beneficial trend, as the original cyst size with the accompanying distortion of cardiac structures would potentially have required a more complex intracardiac surgical reconstruction. This staged approach led to the successful management of our patient, who was subjected to a technically far simpler operation than the one originally believed to be necessary. While the incidence of rupture of inactive hydatid cysts is low, their potential to cause malignant ventricular arrhythmias is unaffected by cyst activity. Surgical excision eliminates the risk of recurrent ventricular arrhythmia [11–13].

Echinococcosis of the heart is a rare but potentially lethal infection. Echocardiography and CMR are not only useful in establishing the diagnosis, but also in defining disease activity.

While only surgery is curative, presurgical medical management can limit cyst growth, but, in rare cases, it may also lead to regression in size. Individual timing of the surgery provides the best chance for optimal outcomes. Patients with echinoccocal cysts harbor the potential for malignant arrhythmia development and inflow/outflow obstructions, warranting a high level of suspicion as to these events.

## Additional file

Additional file 1: Abbreviated view of patient diagnostic and treatment course. Timeline of the clinical picture, diagnostics and treatment. (Black boxes – Relevant past medical history and final resolution of the case. Green boxes – Clinical picture and diagnostic evaluations of the current illness. Red boxes – Medical therapy and interventions applied. TTE = transthoracic echocardiography, CT = computed tomography, MR = magnetic resonance). (DOCX 35 kb)

## Abbreviations

CMR: Cardiac magnetic resonance
IVS: Interventricular septum
LV: Left ventricle
RV: Right ventricle
TTE: Transthoracic echocardiography
