# Supplementary material for: Staged management of a giant cardiac hydatid cyst: a case report
Source: BMC Infect Dis. 2018 Dec 27;18:694. doi: 10.1186/s12879-018-3599-2 (PMC6307286; doi:10.1186/s12879-018-3599-2)
Supplement: Supplementary file 1 — Abbreviated view of patient diagnostic and treatment course. Timeline of the clinical picture, diagnostics and treatment. (Black boxes – Relevant past medical history and final resolution of the case. Green boxes – Clinical picture and diagnostic evaluations of the current illness. Red boxes – Medical therapy and interventions applied. TTE = transthoracic echocardiography, CT = computed tomography, MR = magnetic resonance). (DOCX 35 kb) [file 12879_2018_3599_MOESM1_ESM.docx]

- Degradation and downsizing of cyst enabled cardiac surgery
- Praziquantel was added in therapy in dose of 50 mg/kg divided in 3 doses per day during 14 days

June 26^th^ 2017

Surgical excision of hepatic hydatid cyst

Dec 2017 - Last follow up visit- patient asymptomatic without signs of disease recurrence

- Medical therapy was initiated (albendazole 15 mg/kg) due to technical challenges of cardiac operation
- Bisoprolol and amiodarone were started for cardiac arrhythmia
- CT scan revealed hydatid cyst in liver
- Unremarkable MR of brain
- CMR confirmed TTE findings
- Multiple non sustained ventricular tachycardia detected
- ELISA and Western blot positive for echinococcosis

Hospitalization

- Unremarkable physical examination
- Unremarkable laboratory findings
- TTE exam revealed hydatid cyst of interventricular septum

Sep 2016 - Patient started to feel fatigue and weakness in exertion, along with palpitations and blurred vision

Apr 26^th^
2017

Unremarkable postoperative TEE with normal size and function of both ventricles with preserved continuity of interventricular septum

Successful cardio-surgical excision of hydatid cyst

Continuation of albendazole therapy for 3 cycles of 28 days with 14 day pause between them

May 15^th^ 2017

May 3^rd^ 2017

Patient experienced loss of consciousness due to ventricular tachycardia

- Serial TTE revealed degradation and downsizing of cardiac hydatid cyst confirmed with CMR
- No signs of dissemination were observed on MR of head and CT of thorax and abdomen

Feb 22-28^th^
2017

Feb 21^st^ 2017
